# Assessing the quality of supplementary sensory feedback using the crossmodal congruency task

**DOI:** 10.1038/s41598-018-24560-3

**Published:** 2018-04-18

**Authors:** Daniel Blustein, Adam Wilson, Jon Sensinger

**Affiliations:** 1 Institute of Biomedical Engineering, University of New Brunswick, Fredericton, NB Canada; 2 Department of Electrical and Computer Engineering, University of New Brunswick, Fredericton, NB Canada

## Abstract

Advanced neural interfaces show promise in making prosthetic limbs more biomimetic and ultimately more intuitive and useful for patients. However, approaches to assess these emerging technologies are limited in scope and the insight they provide. When outfitting a prosthesis with a feedback system, such as a peripheral nerve interface, it would be helpful to quantify its physiological correspondence, i.e. how well the prosthesis feedback mimics the perceived feedback in an intact limb. Here we present an approach to quantify this aspect of feedback quality using the crossmodal congruency effect (CCE) task. We show that CCE scores are sensitive to feedback modality, an important characteristic for assessment purposes, but are confounded by the spatial separation between the expected and perceived location of a stimulus. Using data collected from 60 able-bodied participants trained to control a bypass prosthesis, we present a model that results in adjusted-CCE scores that are unaffected by percept misalignment which may result from imprecise neural stimulation. The adjusted-CCE score serves as a proxy for a feedback modality’s physiological correspondence or ‘naturalness’. This quantification approach gives researchers a tool to assess an aspect of emerging augmented feedback systems that is not measurable with current motor assessments.

## Introduction

The performance of clinically-available upper-limb prostheses has been partly hindered by a lack of intuitive and useful feedback^1,2^. Direct neural or cortical stimulation to convey force or other feedback to a user controlling a prosthetic hand may lead to improved systems that better mimic the dynamics of the intact human hand. Peripheral nerve interfaces (PNIs) with bidirectional communication between device and body have been shown effective in controlled settings^3–6^. Advances in feedback applied via spinal or cortical stimulation also show promise to drive forward prosthetic technologies^7–10^. Efforts to improve long term viability, once a concern for such invasive interfaces with the nervous system, have also advanced using wireless signal transmission^11^, osseointegration approaches^12^, and stable electrode designs^13^. While the promise of emerging prosthetic feedback systems is apparent, the development of feedback assessment tools has lagged these emerging technologies.

Traditional performance-based movement assessments may not capture the overall quality of a novel feedback modality. Standard clinical motor assessments such as the Box and Blocks Test^14^, the Nine Hole Peg Test^15^, the Southampton Hand Assessment Procedure^16^ and the Assessment of Capacity for Myoelectric Control^17^ focus on quantifying motor performance but do not provide insight into other potential factors of particular feedback systems. Emerging feedback systems may provide intrinsic value beyond motor performance gains. Evidence suggests that user-trusted feedback can lead to aspects of incorporation and embodiment^18^, that could improve prosthesis acceptance^2^, reduce phantom pain^19,20^ or provide other benefits that would not be detected by traditional motor assessments^21^.

Emerging prosthetic feedback studies have relied on qualitative subjective user descriptions of feedback quality^4,22^. Users report the location and sensation of the applied feedback and the quality of that feedback is inferred. For example, in one study participants described sensations in terms of pressure, tingle, vibration, or light moving touch^4^. As stimulation intensity was varied, one subject described the sensation as changing from “tingly” to “as natural as can be”^4^. Some conclusions are clearly justifiable, for example, self-described “natural, non-tingling feedback” is presumably better than “uncomfortable, deep, dull vibration”^4^. But not all self-reported sensory descriptions are as easy to interpret. Recent focus has shifted to make these qualitative observations more reliable and quantifiable^23^. When a user perceives touch through a feedback system, how closely does that percept match the touch sensation experienced with intact anatomy? Unnatural feedback may be difficult to interpret, or even painful^24^. In this study we have sought to objectively quantify the physiological correspondence, or naturalness, of supplementary sensory feedback modalities.

**Figure 1:**
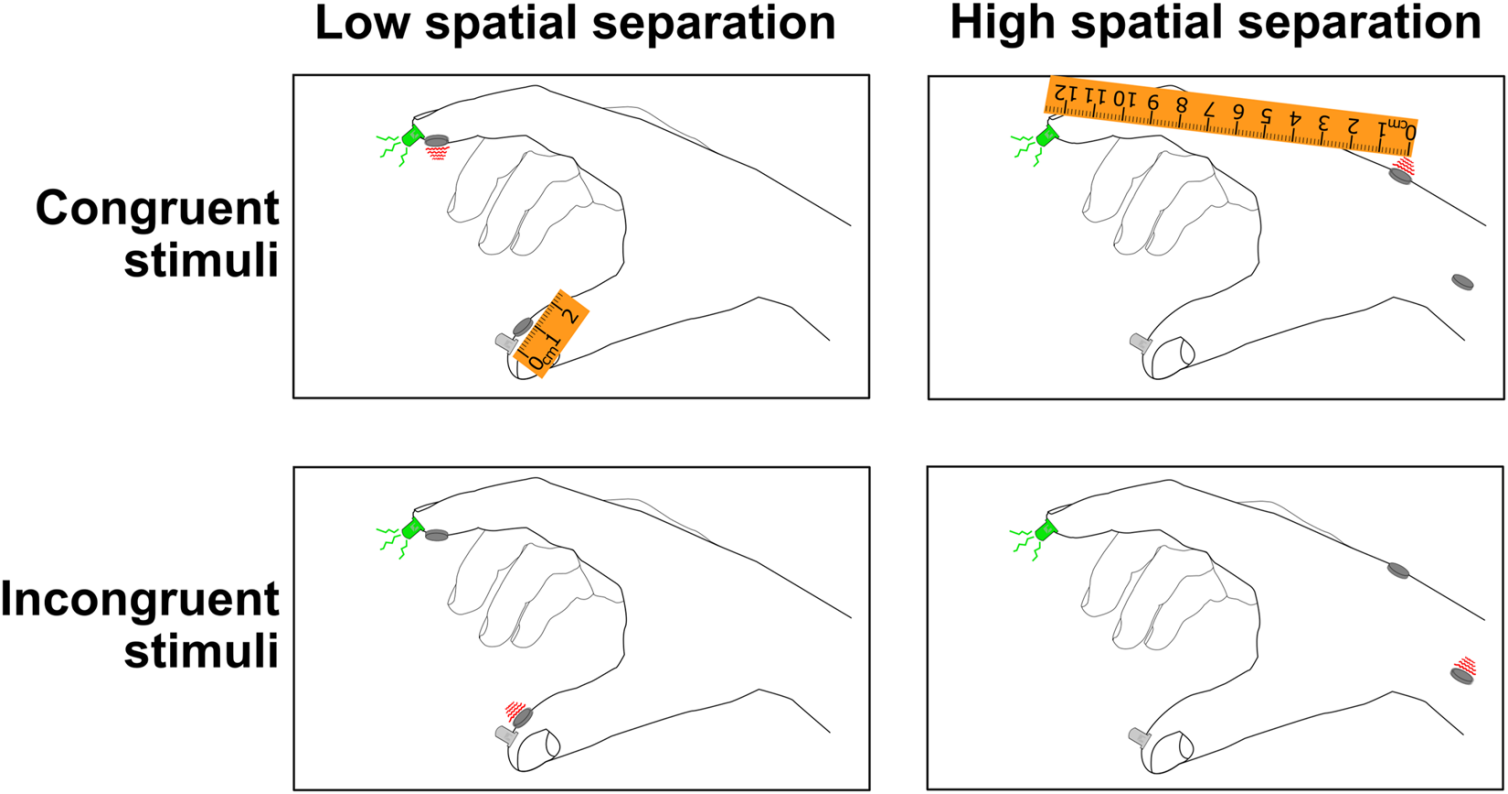
Crossmodal congruency effect framework. Participants rapidly select the target feedback (e.g. vibration shown in red). The target feedback is presented concurrently with distractor visual feedback (green LED). Feedback can be presented congruently, i.e. visual and target feedback collocated in the top row, or incongruently with mismatched visual and target feedback. The crossmodal congruency effect (CCE) score is the difference between the response time to congruent and incongruent stimuli. The cross-modal effect can also manifest as an elevated error rate during incongruent trials^25^. Spatial separation, the physical distance between the two paired sensory percepts, can be varied, and is indicated with the rulers.

Quantifying the quality of a feedback system is the goal of this work but the spatial alignment of the perceived feedback can be a confounding factor. When stimulating the nervous system via a PNI or other brain-machine interface, the perceived location of the feedback may differ from the intended feedback location. For example, a visually-observed contact on a prosthetic fingertip may be felt, as the result of neural stimulation, several centimeters away at the base of the palm (see Fig. 1, top right panel)^22^. This spatial misalignment between actual and expected percept may greatly affect the feedback’s usability. Assessments of feedback quality must consider both the effect of misaligned percepts and the naturalness of the stimulation.

The crossmodal congruency effect (CCE) score provides an objective measure of incorporation of a feedback modality^25–28^. The degree of feedback incorporation, indicated by the CCE score, is affected by the two interacting factors affecting feedback quality: spatial alignment of percepts and physiological correspondence, or naturalness^25,29,30^ (Fig. 1). This metric has been used to quantify incorporation in the rubber hand illusion^28,31^ and shows promise for assessment of neuroprosthetics^32^. In this study we aim to remove the spatial bias from the CCE score to arrive at an adjusted CCE score that is a proxy for physiological correspondence.

The CCE task is an established psychophysics protocol that involves the speeded discrimination of target stimuli^25^. Participants are presented with a target stimulus in one of two locations. Concurrent distractor feedback in the form of an illuminated LED is presented in one of two alignments: 1. *Congruent*, the illuminated LED is on the same side as the target feedback (Fig. 1, top row); or 2. *Incongruent*, the illuminated LED is on the opposite side of the target feedback (Fig. 1, bottom row). The participant is asked to rapidly select the location of the target feedback with one of two foot pedals that each correspond to one “side” of the target feedback. In one of our implementations, the left and right foot pedals correspond to vibratory target feedback on the thumb and index finger, respectively (Fig. 1, left column).

The CCE score is calculated as the difference between the reaction time to incongruent stimuli and the reaction time to congruent stimuli. An increase in the incongruent reaction time relative to the congruent reaction time is indicative of longer cognitive processing times required to ignore the distractor LED and select the target feedback with incongruent stimuli. Incongruent reaction time, and therefore CCE score, is maximized when the conditioned response to the target feedback is strongest, i.e. the feedback modality is highly incorporated into a person’s body schema^33,34^. As the target feedback becomes less natural feeling, it is more poorly incorporated, resulting in a weaker conditioned response and lower CCE scores^28^. CCE is a useful measure, however, its value as a direct measure of physiological correspondence is conflated by its dependency on spatial co-location^25^. Here we show that the spatial separation factor can be removed from the CCE score, enabling a measurement of physiological correspondence that is independent of spatial co-location.

We completed a comprehensive study of 60 able-bodied participants controlling a bypass prosthesis under different feedback conditions. We found that incorporation after training, as measured by CCE score, changes with feedback modality (i.e. physiological correspondence). After extended training, CCE score increased as the spatial separation between expected and perceived feedback decreased. The model we generated from these data provides an adjusted CCE score with the effect of spatial separation removed. This provides a bias-free quantification of physiological correspondence, or naturalness. Since the target feedback applied in the CCE task is analogous to the feedback applied via a PNI with a sensorized prosthesis, this adjusted CCE score assessment can be used to quantify an aspect of the naturalness of a prosthetic feedback system. The approach we present provides an important first step towards quantitatively measuring the degree to which feedback actually feels physiologically accurate.

## Results

**Figure 2:**
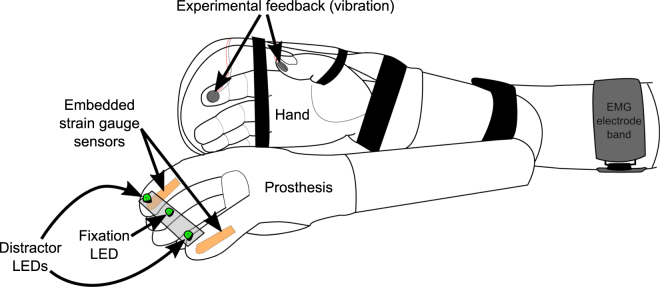
Experimental setup. Able-bodied participants donned a bypass prosthesis^36^ during a training phase that immediately proceeded the CCE score assessment. During the CCE protocol, participants were provided target feedback (e.g. vibration) coupled with visual feedback via the distractor LEDs. Participants were asked to respond as quickly as possible, with foot pedals, to select the location (left vs. right) of the target feedback. See Methods for more details on the protocol. During training, participants controlled the bypass prosthesis with electromyographic signals to move mechanical eggs over a barrier. The sensorized prosthesis could detect force applied to the thumb and the index finger via embedded strain gauge sensors and provide proportional feedback to the user conveyed as vibration, electrical stimulation or skin deformation. During training and testing the intact hand and harness are covered with black fabric.

To determine if different modalities of feedback provided to a person could be incorporated, we measured the CCE score^25,35^ of 60 able-bodied individuals after training with a bypass prosthesis^36^ (Fig. 2). Participants trained using the bypass prosthesis to move mechanical eggs with one of three feedback modalities: vibration, electrical stimulation or skin deformation^36^. Training duration (short vs. extended) and spatial separation between the expected feedback on the fingertip contact point and the perceived feedback (matched on fingertip vs. >12 cm away) were also varied (see Supplementary Table S1 for all experimental conditions). We first show that CCE is a useful measure of physiological correspondence, i.e. it is sensitive to changes in feedback modality. We then demonstrate that the CCE is affected by changes in spatial separation. Then we generate a regression model that can remove the effect of spatial separation from the CCE score, resulting in an adjusted CCE score that captures an unbiased measure of physiological correspondence.

**Figure 3:**
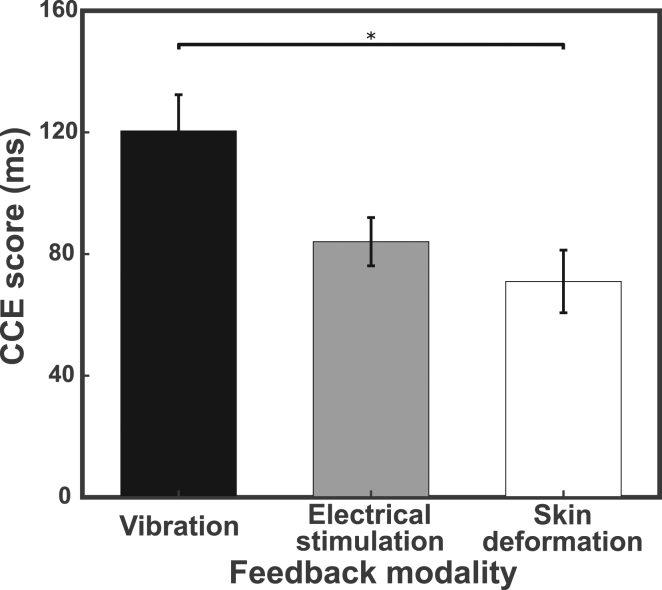
Feedback modality affects CCE score. Means and standard error plotted for 20 participants in each feedback modality group. Statistical significance verified using three-way ANOVA with Bonferroni post-hoc comparison. *p < 0.05.

We observed a main effect of feedback modality on CCE score (Fig. 3). A three-way ANOVA with CCE score as the dependent variable and three categorical independent variables representing spatial separation, training level and the three feedback modalities resulted in a statistically significant effect of feedback (F(2,48) = 6.015, p = 0.0047, ω = 0.28). None of the interaction terms between independent variables were statistically significant (p > 0.05). Bonferroni post-hoc tests showed that the CCE score was significantly higher for vibration feedback [µ = 120 ms ± 53] compared to skin deformation feedback [µ = 71 ms ± 46] (p = 0.0048) and non-significantly higher than electrical stimulation [µ = 84 ms ± 36] (p = 0.052). A higher CCE score indicates a higher level of incorporation for that feedback modality^25^. The trends observed with data binned for each modality are also seen with the CCE scores of each of the 12 treatment groups (Supplementary Fig. S1). Feedback modalities differ in their level of physiological correspondence to intact biological feedback. Changes in the provided feedback modality thus represent a change in physiological correspondence, a manipulation that had a significant effect on the measured CCE score.

**Figure 4:**
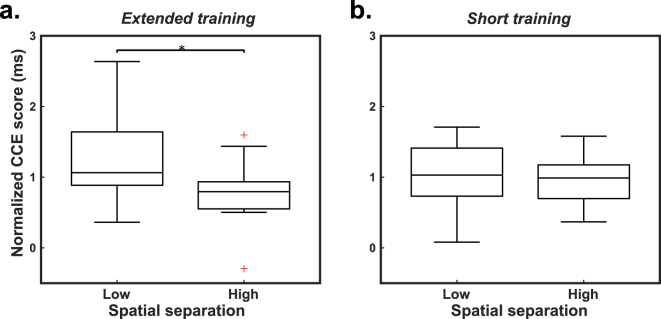
Spatial separation affects CCE score after training. (**a**) CCE score decreased with increased spatial separation for extended-training participants. (**b**) There was no observed effect of spatial separation on CCE score after short periods of training. Statistical significance tested using unpaired two-sample equal variance t-tests: *p < 0.05. Reported results are normalized to the group mean within each feedback type to account for global differences across modalities.

The spatial separation between perceived and expected feedback affects CCE score, but only after extended training (Fig. 4, Supplementary Fig. S1). To isolate the effect of spatial separation from the differences in CCE score observed across modalities (Fig. 3), we normalize CCE scores within each modality and combine normalized scores into one group for comparison (Fig. 4). Within each modality the same trend is observed: after extended training, raw CCE scores are higher with low spatial separation than with high spatial separation (Supplementary Fig. S1). In participants with extended training periods, there was a significant effect of spatial separation on normalized CCE score (unpaired t-test, 2-tailed, p = 0.035) (Fig. 4a). CCE scores decreased as spatial separation increased, indicating that incorporation diminished as the perceived feedback did not align with the expected feedback location. Spatial separation appeared to have no effect on CCE score in participants with a short training period (unpaired t-test, 2-tailed, p = 0.88) (Fig. 4b). Short training involved 50 minutes of practice moving mechanical eggs and extended-training lasted 80 minutes. Since spatial separation only had a significant effect on the measured CCE score after extended training, we do not include the short training data in further analysis.

Given that CCE score is an indicator of physiological correspondence, but spatial separation has a biasing effect on the metric, we attempted to fit a model to calculate an adjusted CCE score. The adjusted CCE score, the CCE_A_, would be unbiased by the confound of spatial separation and solely represent the physiological correspondence of the provided feedback. To approximate the baseline adjusted CCE score (CCE_A_) for each feedback modality, we used the average CCE score for all treatment groups within that modality. This allowed for the conversion of the categorical feedback modalities to a continuous scale. Since we established that CCE score is biased by spatial separation, we used a corrective model to remove this influence. We first fit a multiple linear regression to the data from the 30 extended-training participants (F(2,27) = 4.93, p = 0.015, R^2^ = 0.27). The model can be expressed as

1 $$CCE={B}_{0}(CCEadjusted)+{B}_{1}(SpatialSeparation)+{B}_{2}$$

where B_0_ = 0.98 (p = 0.028), B_1_ = −37.6 (p = 0.043) and B_2_ = 22.85 (p = 0.58). The spatial separation term was defined as zero for spatial separations of 3.0 cm or less, and one for spatial separations greater than 12.0 cm. Note that the intercept coefficient B_2_ is not statistically different from zero but was included so that the linear model could be used with low CCE scores under high spatial separation conditions. Given measured values for CCE score and spatial separation, the CCE_A_ of a feedback system can be estimated by rearranging Equation 1 as

2 $$CCEadjusted=\frac{CCE+37.6\,\ast \,SpatialSeparation-22.8}{22.8}$$

**Figure 5:**
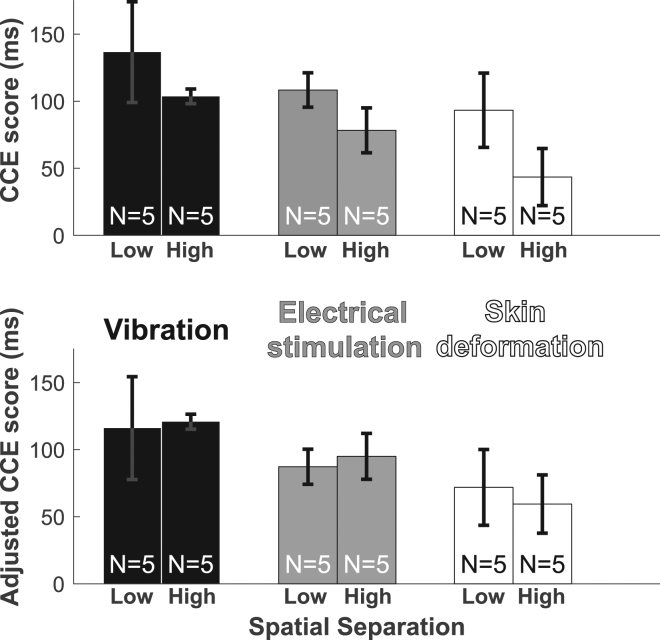
Adjusted CCE score is not affected by spatial separation. Top panel. CCE score means and standard error for the extended-training participants. For each modality, the CCE score is lower for the high spatial separation group. Bottom panel. CCE_A_ results for the extended-training participants. The effect of the spatial separation observed in the CCE score results is not present.

This equation was used to calculate the CCE_A_ for all extended-training participants (Supplementary Fig. S2). The spatial separation bias on CCE score (Fig. 4a) was observed as trends within the extended-training results for each modality (Fig. 5, top panel). This bias was not present when observing CCE_A_ results of the same participants calculated using Equation 2 (Fig. 5, bottom panel), supporting the model’s ability to account for the effect of spatial separation.

We further analyzed the data to investigate possible explanations for the low CCE score observed with skin deformation feedback. The low CCE score could not be attributed to the latency of the skin deformation feedback application (Supplementary Data S1). Additionally, we observed no significant trend between motor performance (movement success rate during training) and CCE score (Supplementary Fig. S3).

## Discussion

We have demonstrated that CCE scores can be used to assess feedback quality. We collected CCE scores from 60 able-bodied participants using a bypass prosthesis with different feedback systems. From the results we developed a corrective model to output adjusted CCE scores, unaffected by spatial separation biases, that are a proxy for physiological correspondence. The adjusted CCE score can be used to assess other novel sensory feedback systems, such as patients with peripheral nerve interfaces or cortical stimulators. The psychophysics-based technique we have presented fills a need for more informative assessment of advanced feedback systems.

**Figure 6:**
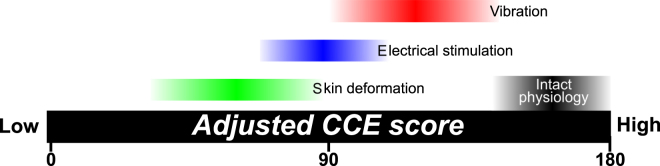
Physiological correspondence benchmark scale. Benchmark data for different feedback modalities to allow for comparison and contextualization of results from the assessment of novel feedback systems. Maximum CCE scores using intact physiology and no spatial separation are indicated^35^.

Given the adjusted CCE quantification model (equation (2)) and the extended-training data we collected, we provide a scale to contextualize CCE_A_ scores (Fig. 6). Researchers who measure the CCE score and spatial separation of a feedback system can calculate the CCE_A_ using Equation 2. When assessing the physiological correspondence of novel feedback modalities, researchers can use the scale in Fig. 6 as a benchmark for the analysis of results. For example, if a feedback modality’s assessed CCE_A_ is 130, then the feedback would have a similar level of correspondence to vibration feedback. This example feedback modality would have a high level of physiological correspondence.

Our results support previous findings^25,35^ showing that CCE score is influenced by feedback modality and spatial separation, i.e. the offset between the target feedback and the distractor visual feedback. Although advanced feedback systems strive to minimize spatial separation, the imprecision of neural stimulation makes the generation of perfectly-aligned percepts quite difficult. A high spatial separation will affect the incorporation of the feedback, resulting in a lower CCE score^25^. Additionally, the dynamics and timing of feedback can affect a user’s subjective assessment of its “naturalness”^4^. Researchers often strive to elicit natural feeling percepts with experimental feedback systems. But to our knowledge we have not seen any attempts at objectively quantifying this “naturalness” sensation. We use the CCE_A_ score to capture the degree of physiological correspondence, i.e. how well a feedback modality mimics the feedback experienced with intact anatomy, as a proxy for “naturalness”. Since CCE score and spatial separation are easily measurable, we can use the relationship between these three variables to quantify the CCE_A_ of supplementary sensory feedback.

One potential limitation of the model we developed is that CCE scores may be affected by additional factors besides physiological correspondence, spatial separation and training. We did not factor in the effect of fatigue, time of day, baseline reaction time, and feedback characteristics such as latency, consistency and dynamics. Variability in feedback characteristics were limited by our use of a real-time embedded system to provide feedback with correspondingly low latency (<30 ms for vibration and skin deformation). However, the method used to calibrate the skin deformation feedback may have led to variable levels of incorporation. For example, the haptic tactor starting position was not clearly visible in the experimental setup and for some participants it may have been in contact with the skin or arm hair at zero-force levels. Although we could not eliminate the effects of all potential factors affecting CCE score, the most significant factors seem to be physiological correspondence, spatial separation and training as evidenced by their effects on our CCE results and by previous observations^25,37^.

CCE scores were affected by training duration: spatial separation only affected CCE score in the extended-training participants (Fig. 4). It seems that the short-training participants did not have enough exposure to the feedback to reach a maximum level of incorporation, a qualitative finding observed elsewhere although on different timescales^38^. Therefore, to generate the CCE adjustment model we used only extended-training data. The extended-training group had 80 minutes of practice with the feedback system, which is less training than would be typical for patients using this assessment. A patient with a novel feedback system will often complete a take-home trial, wearing a device for days to weeks, before running this assessment. Therefore, we consider only extended-training data and define the 80-minute duration as the minimum exposure necessary for this assessment to be effective. We expect the effect of training to plateau and that the model should be applicable to longer training times; nevertheless, this should be verified with an additional study.

Although the model’s goodness-of-fit seems low (R^2^ = 0.27), this is a consequence of the noisiness of human psychophysical data and it can still be used to assess supplementary feedback quality. However, the variability of CCE scores across individuals may make one-to-one comparison between individuals difficult. We based our model development on a population level analysis that, while statistically valid, could lead to misinterpretation of a single CCE result. Therefore, we recommend comparisons of CCE scores from the same individual across different feedback modalities or with different training periods. Alternatively, when data from several individuals are available, a population-level comparison can be made. A power analysis should be run to determine the number of individuals necessary to detect a certain level of improvement supplied by a novel feedback system^39^. To detect the maximum intergroup difference observed in our study (93 ms), and assuming the observed overall variability across all 60 participants (SD = 46 ms, normalized to the mean within each group), three individuals would be needed to achieve a statistical power of 0.8 at a confidence level of 95%. Increasing the number of individuals tested would allow for smaller CCE score differences to be detected. For example, to detect half of the maximum difference observed (δ = 47 ms), eight individuals should be tested. The exact number of individuals required depends on the CCE score variability that will vary depending on the feedback system and patient population. In either the repeat-individual testing approach or the small-group population analysis, a CCE task learning effect must be considered when scheduling test sessions^35^.

The low CCE score observed for skin deformation feedback (Fig. 3) was an unexpected result. Originally we hypothesized that using skin deformation feedback to represent the grasp force of the training movements would result in the highest level of incorporation. Skin deformation more closely resembles the physical activation of a grasping force compared to vibration and electrical stimulation. However, skin deformation feedback resulted in the lowest CCE score compared to electrical stimulation and vibration, a statistically significant result that does not appear to be the result of noise or random fluctuation. We confirmed that the poor incorporation of skin deformation was not due to mediocre actuation as latency results were consistent across feedback modalities. In some participants the tactor may have been in contact with the skin at a zero-force level. Variable skin contact would result in variable perception across participants as the discrete initial skin contact has been shown to be important in improving feedback effectiveness^40^. The low incorporation of skin deformation feedback could alternatively be explained by long-term depression of afferents due to repeated stimulation or slipping actuators; both explanations would be supported by an observed change in detection threshold over the course of the experiment (see Supplementary Data S1). A future study is planned to combine the CCE score assessment with an outcome metric that assesses feedback uncertainty to more carefully characterize the utility of the skin deformation feedback^41^.

Participants performed well using skin deformation feedback (Supplementary Fig. S3), but we still observed poor incorporation. There did not seem to be a trend between motor performance, measured as the percentage of mechanical eggs moved during the training phase, and CCE score, implying that performance and incorporation are distinct concepts. An individual’s quantifiable motor performance may be inflated through the adoption of alternative strategies and compensatory movements^42,43^. Further, motor performance does not necessarily correspond to other important aspects of prosthesis use such as device acceptance^18^, phantom pain reduction^19,20^ or cognitive burdens^21^. Therefore, clinical movement assessments relying only on motor performance may not be suitable to analyze the performance of advanced feedback systems. These assessments also suffer from other limitations such as a reliance on movement timing and variability introduced by rater subjectivity^44^. Available motor assessments may be sufficient to monitor clinical progress but no single outcome metric captures all relevant performance information^45^. The CCE-based assessment and supporting model we have presented could augment the battery of performance-based assessments currently in use to provide more detailed insight into supplementary feedback system performance.

We have presented a data-driven model approach that can remove the spatial separation bias from the CCE score to provide an indicator of the physiological correspondence of a feedback modality. This approach represents a way to provide more informative assessment of prosthetic feedback systems. Further steps will require the clinical validation of this assessment tool in patient populations, such as amputees outfitted with peripheral nerve feedback systems. Novel feedback systems for amputees require novel assessment tools; this work provides an advanced outcome metric to fill that need.

## Methods

### Participant recruitment

Participants were recruited by word-of-mouth and provided informed consent under the guidelines and approval of University of New Brunswick’s Research Ethics Board. All methods were performed in accordance with relevant ethics guidelines and regulations. Sixty volunteer participants completed the study [mean age = 31.9 yrs, range = 18–76 yrs, 22 female, 5 left-handed]. Participants were randomly assigned to a treatment condition which specified feedback modality [vibration, electrical stimulation or skin deformation], training duration [short or extended], and spatial separation between visual and target feedback [low or high]. There were ten treatment groups and each participant completed training and CCE assessment for one modality at a given spatial separation and for a particular training duration.

### Bypass prosthesis

Participants first trained using a bypass prosthesis with myoelectric control and embedded force sensors in the thumb and index finger that proportionally drove feedback intensity. The bypass hardware is described in detail elsewhere^36^. Each participant only received the assigned feedback modality at the assigned spatial separation condition.

### Feedback implementation

Skin deformation feedback was applied using linear mechanotactile haptic tactors attached to the subject (design courtesy of the University of Alberta^46^). Each tactor used a rack and pinion gear system to convert rotational motion generated by a servo motor (HiTec, HS-35HD) to linear motion that was applied to the subject’s skin via an 8 mm diameter domed head. Measured force from the sensorized prosthetic hand (custom retrofitting of Ottobock MyoHand VariPlus Speed by HDT Global) was mapped to servo displacement. Zero force was mapped to a displacement that was a step below the minimum detectable level. The maximum displacement was based on the current draw of the servos and limited to approximately 100 mA. This level was selected to keep the actuation at a level below which the plastic rack and pinion system would not slip. During the training phase, the tactor displacements were proportionally controlled to match the measured forces on the thumb and index finger of the instrumented prosthetic hand. During the CCE score assessment, the tactors were displaced to approximately 20–25% of the maximum experienced during the training phase.

Vibration feedback was provided by two 10 mm linear resonant actuators (LRAs: Precision Microdrives, C10–100) taped to the skin with medical tape (3 M, Micropore). During the training phase, the LRAs were proportionally controlled to correspond to the measured forces on the thumb and index finger of the instrumented prosthetic hand. During the CCE score assessment, the stimuli were set to approximately 20–25% of the maximum intensity experienced during the training phase.

Electrical stimulation was provided by a 2-channel TENS electro-stimulator (Proactive, Pulse). The device was modified such that the electrical stimulation intensity could be controlled with isolated analog outputs from a myRIO embedded hardware system (National Instruments). During the training phase, the stimulator outputs were proportionally controlled to produce paresthetic sensations that corresponded to the measured forces on the thumb and index finger of the instrumented prosthetic hand. During CCE score assessment, the stimuli were set to the maximum intensity experienced during the training phase. The protocol for electrical stimulation was modified compared to the other modalities to limit participant discomfort and avoid painful percepts.

### Feedback thresholds

Feedback detection thresholds were measured for each subject to calibrate the stimulation before the training phase. The stimulus intensity was slowly increased until the subject indicated that the stimulation was felt. This was repeated three times and the lowest reported stimulus level was used to set the range of stimulus. A proportional mapping was used to convert the hand’s force detection range to the subject’s stimulus detection range. The low end of the force detection range was set slightly below the reported detection threshold (~1% PWM duty cycle decrease for vibration and skin deformation feedback; ~10 mV decrease for electrical stimulation). The maximum feedback was set to correspond to 1.2× the breaking threshold of the heaviest egg (19.4 N). The maximum stimulus level was set based on the type of feedback. The LRAs were set to their maximum achievable intensity for the maximum stimulus level. The electrical stimulus maximum level was set based on the subject’s comfort and to avoid muscle twitch. The feedback detection threshold was measured again after the training phase, immediately preceding CCE score assessment.

### Spatial separation

For feedback with low spatial separation, the target feedback was applied at the fingertip to match the visually-observed contact point on the prosthetic hand. For feedback with high spatial separation, the actuators were attached to the wrist for skin deformation and vibration. For the electrical stimulation low spatial separation group, the self-adhesive electrical stimulation pads were wrapped around the index finger or thumb. Electrical stimulation on the wrist interfered with EMG control signals so for the high spatial separation group the pads were placed on the back of the hand near the major knuckles of the index finger and thumb.

### Electromyographic control

During training, the one-degree-of-freedom prosthetic hand of the bypass was controlled with a Complete Control (Coapt) pattern recognition system. Participants trained hand open and close control using isometric wrist flexor and wrist extensor muscle contractions using the commercial software provided by Coapt.

### Training protocol

**Figure 7:**
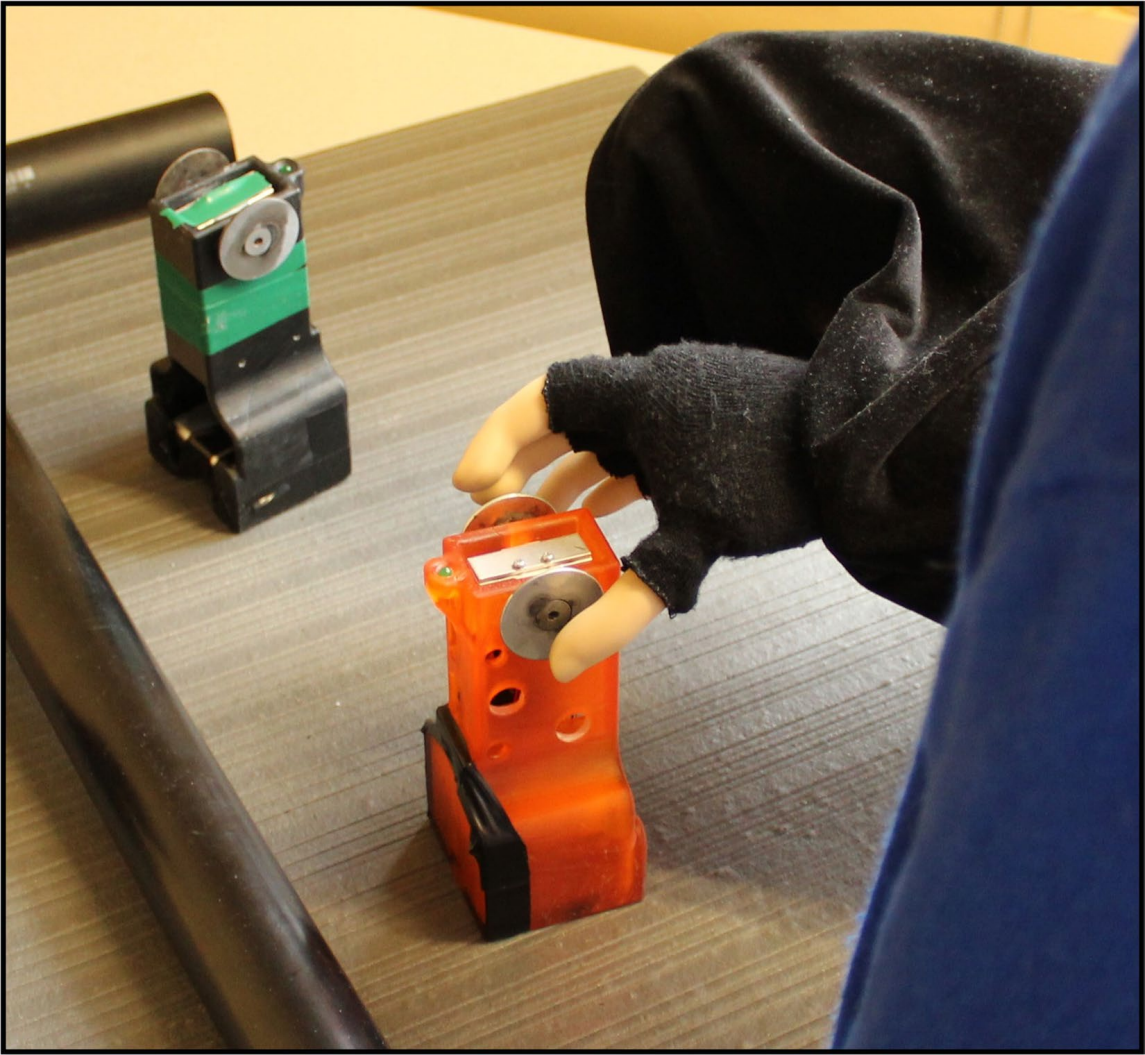
Variable weight mechanical eggs were moved during training periods. Load cells on eggs detected grasp force and simulated an egg breakage with a light cue when a threshold was exceeded.

Participants in the short-training group completed five training sessions, each lasting ≤10 minutes with 10-minute intervening breaks, for 50 minutes of total training. Extended-training participants completed eight sessions for 80 minutes of total training. In each training session participants attempted to move instrumented mechanical eggs of three different weights and “breaking” thresholds over a 5 cm high barrier (Fig. 7). The lightest egg weighed 2.78 N with a breaking threshold of 6.84 N. The medium-weight egg weighed 5.45 N with a breaking threshold of 10.52 N. The heaviest egg weighed 9.55 N with a breaking threshold of 16.19 N. Each session ended after 100 movement attempts or ten minutes, whichever occurred first. Successful and unsuccessful movements were recorded manually by the experimenter. When too much force was applied to the mechanical egg, an on-egg LED would illuminate to indicate a broken egg. After breaking a mechanical egg, the subject had to release the egg and restart the movement. Participants wore earplugs and over-ear noise-canceling headphones playing Brownian noise to mask actuator and background noise.

### CCE assessment

Following the bypass training, the participant’s CCE score was assessed^25^ using a modified protocol^35^. Participants were asked to make speeded responses to select the location of target feedback presented randomly to one of two locations (see Fig. 1) using foot pedals. Left and right foot pedals corresponded to thumb and finger feedback locations, or the spatially separated corollaries of those locations. Participants focused on a centrally-located fixation LED that illuminated at the start of each trial. After 1000 ms, the left or right distractor LED attached to the prosthesis would illuminate concurrent with target feedback applied to the participant’s hand or arm at the left or right location (see Fig. 2). Four conditions were possible, congruent and incongruent stimuli for left or right target feedback, and were presented in a random order over sequential trials. Visual and target feedback was provided for 250 ms and participants were asked to respond as quickly as possible to select the target feedback location.

Participants first completed three familiarization sessions of ten trials each and then four assessment blocks of 64 trials each. Participants were seated beside a height adjustable table that was set to a comfortable height. A pillow was placed under each subject’s arm to ensure forces and vibrations were not transmitted through the table surface. CCE score for each block was computed as mean incongruent time minus mean congruent time. The overall CCE score was calculated as the mean of the scores from the four blocks.

### Statistical analysis

Statistical analysis was run using IBM’s SPSS Statistics and MATLAB software. A multi-way ANOVA was run with dummy categorical variables used to represent feedback modality, spatial separation and training level. Effect size was calculated as ω^2^ and reported as the square root, ω^47^. For the linear regression analysis (see equation (1)), only extended-training data were used. CCE score was the dependent variable and Feedback Location and baseline CCE score were the independent variables. The Feedback Location variable was set to either zero (distances of 0 to 3 cm, at or near the fingertips) or one (distances of more than 12 cm from finger tips, on the wrist or back of hand). Baseline CCE score for each modality was set as the mean CCE score for a particular feedback type (71 for skin deformation feedback, 120.5 for vibration, 84.1 for electrical stimulation).

### Data availability

All data are available in the Dataset 1 file that accompanies this manuscript.

## Electronic supplementary material

Supplementary Information

Dataset 1
