# Author Correction: Assessing the quality of supplementary sensory feedback using the crossmodal congruency task

**DOI:** 10.1038/s41598-018-31647-4

**Published:** 2018-09-12

**Authors:** Daniel Blustein, Adam Wilson, Jon Sensinger

**Affiliations:** 1 Institute of Biomedical Engineering, University of New Brunswick, Fredericton, NB Canada; 2 Department of Electrical and Computer Engineering, University of New Brunswick, Fredericton, NB Canada

Correction to: *Scientific Reports* 10.1038/s41598-018-24560-3, published online 18 April 2018

This Article contains an error in Equation 2.

$$CCEadjusted=\frac{CCE+37.6\ast SpatialSeparation-22.8}{22.8}$$

should read:

$$CCEadjusted=\frac{CCE+37.6\ast SpatialSeparation-22.8}{0.98}$$

