# Supplementary material for: Assessing the quality of supplementary sensory feedback using the crossmodal congruency task
Source: Sci Rep. 2018 Apr 18;8:6203. doi: 10.1038/s41598-018-24560-3 (PMC5906608; doi:10.1038/s41598-018-24560-3)

Supplementary Information

Title: *Assessing the quality of supplementary sensory feedback using the crossmodal congruency task*

Authors: **Daniel Blustein, Adam Wilson, Jon Sensinger**

Supplementary Table S1. Experimental conditions for 60 able-bodied subjects. Spatial separation is marked as Ø for 3cm or less and ✓ for greater than 12cm.

**Feedback modality →**	*Vibration*				*Electrical stimulation*				*Skin deformation*			
**Training level →**	Short		Extended		Short		Extended		Short		Extended	
**Spatial separation →**	Ø	✓	Ø	✓	Ø	✓	Ø	✓	Ø	✓	Ø	✓
**# of subjects →**	5	5	5	5	5	5	5	5	5	5	5	5

Supplementary Figure S1. Mean CCE scores and standard error from all 12 treatment groups.

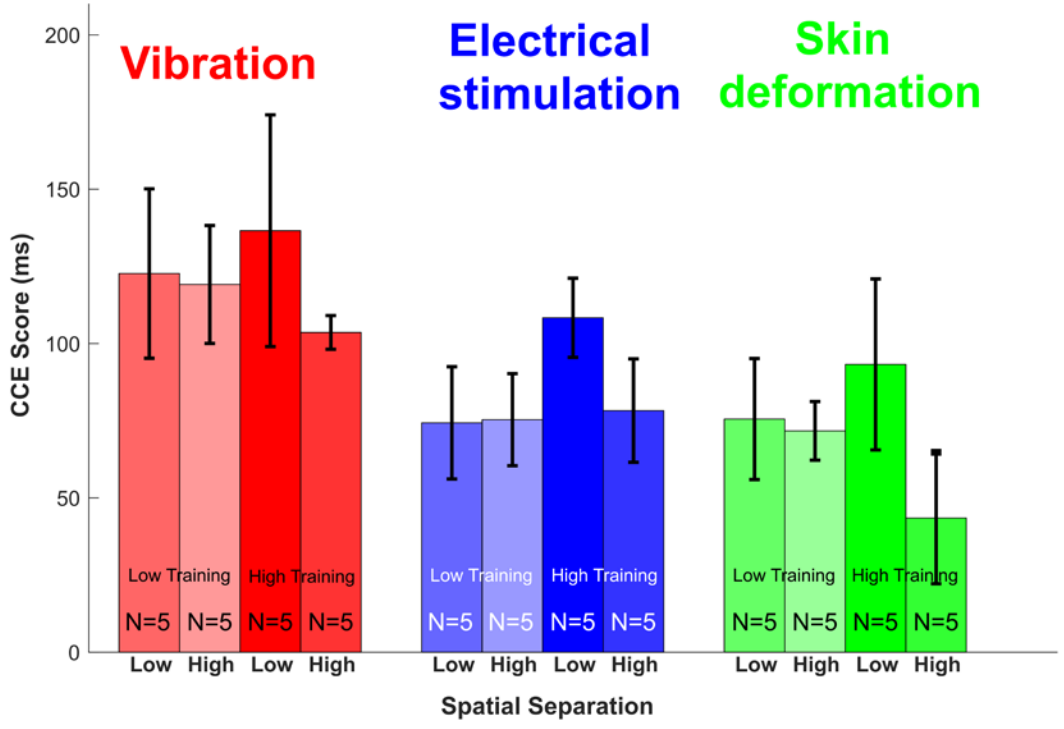

Supplementary Figure S2. Adjusted CCE score comparison. Raw CCE scores are shown for extended training participants with low spatial separation (black points) and high spatial separation results (red points). Adjusted CCE scores (green points) are shown for each spatial separation group. Box plots of the adjacent CCE_A scores are presented.

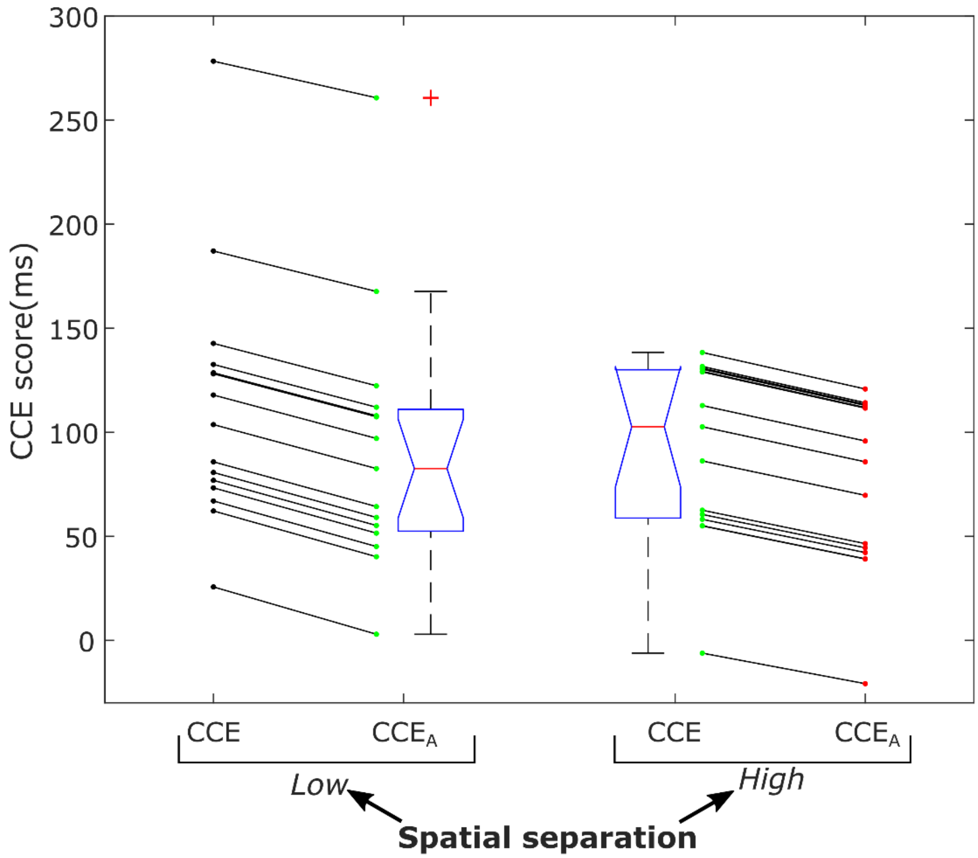

Supplementary Data S1. Additional investigation of low CCE score with skin deformation feedback.

Surprisingly, skin deformation feedback resulted in the lowest mean CCE score, corresponding to the lowest level of incorporation (Fig. 3). We sought to investigate this result by analyzing the success rate of movements during training. Although CCE score tended to increase as the percent of successful egg movements (no drops and no breaks) decreased, this difference was not statistically significant determined with one-way ANOVA ($F(2,57)=2.48$, $p = 0.093$). The raw data show a high variability across subjects (Supplementary Fig. S2).

The incorporation of skin deformation feedback may have been affected by the intensity, timing or other characteristics of the feedback provided. We observed a significant difference (unpaired t-test, $p<0.05$) in the change in detection threshold over the course of training between vibration (0% change) and skin deformation modalities (+51.4% average

change). Detection thresholds were measured at the start of the training phase and at the end of training just before CCE score assessment. The detection threshold of the electrical stimulation feedback was set differently to avoid painful sensations and was not included in this analysis. There were no differences measured between the timing precision of the different feedback modalities. The initial position of the tactor may have affected the effectiveness of the skin deformation feedback. In some subjects the tactor may have been in contact with the skin or arm hair before any sensed force. In future studies, body hair should be shaved and the tactor should be initially positioned to ensure no contact with the subject at zero force levels.

Supplementary Figure S3. CCE score and movement success rate. Although there appears to be an inversely proportional trend, movement success rates were not significantly different across modality (one-way ANOVA; F(2,57)=2.48, p = 0.093).

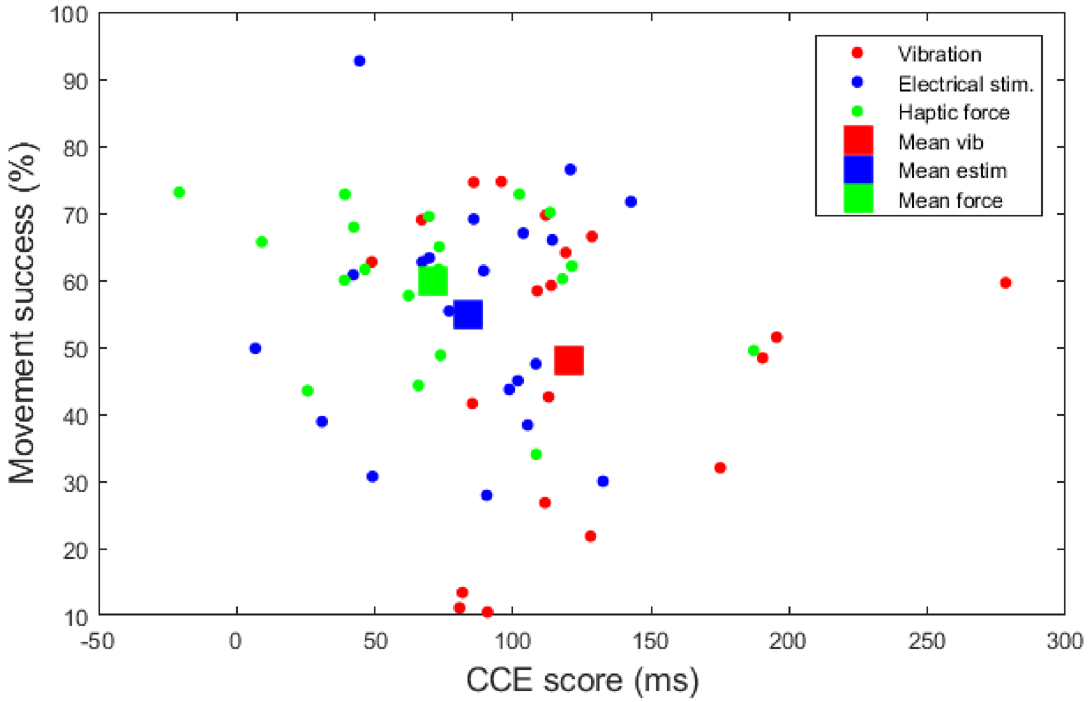

Supplement: Supplementary file 1 — Supplementary Information [file 41598_2018_24560_MOESM1_ESM.pdf]
